# De-implementing low-value care in cancer care delivery: a systematic review

**DOI:** 10.1186/s13012-022-01197-5

**Published:** 2022-03-12

**Authors:** Amir Alishahi Tabriz, Kea Turner, Alecia Clary, Young-Rock Hong, Oliver T. Nguyen, Grace Wei, Rebecca B. Carlson, Sarah A. Birken

**Affiliations:** 1 Department of Health Outcomes and Behavior, Moffitt Cancer Center, 4115 E. Fowler Avenue, Tampa, FL 33617 USA; 2 Department of Oncological Sciences, University of South Florida Morsani College of Medicine, 560 Channelside Dr, Tampa, FL 33602 USA; 3 The Reagan-Udall Foundation for the FDA, 1900 L Street, NW, Suite 835, Washington, DC, 20036 USA; 4 UF Health Cancer Center, Gainesville, FL USA; 5 Department of Health Services Research, Management and Policy, College of Public Health and Health Professions, University of Florida, HPNP Building, Room 3111, Gainesville, FL 32610 USA; 6 Department of Community Health & Family Medicine, University of Florida, P.O. Box 100211, Gainesville, FL 32610 USA; 7 Department of Health Services Administration, University of Alabama at Birmingham, Birmingham, AL USA; 8 Health Sciences Library, The University of North Carolina at Chapel Hill, 335 S. Columbia Street, Chapel Hill, NC 27599 USA; 9 Department of Implementation Science, Wake Forest School of Medicine, 525@Vine Room 5219, Medical Center Boulevard, Winston-Salem, NC 27157 USA

**Keywords:** De-implementation, De-adoption, Low-value care, Low-value service, Cancer, Cancer care delivery, Oncology, Overuse, Choosing wisely

## Abstract

**Background:**

Accumulating evidence suggests that interventions to de-implement low-value services are urgently needed. While medical societies and educational campaigns such as Choosing Wisely have developed several guidelines and recommendations pertaining to low-value care, little is known about interventions that exist to de-implement low-value care in oncology settings. We conducted this review to summarize the literature on interventions to de-implement low-value care in oncology settings.

**Methods:**

We systematically reviewed the published literature in PubMed, Embase, CINAHL Plus, and Scopus from 1 January 1990 to 4 March 2021. We screened the retrieved abstracts for eligibility against inclusion criteria and conducted a full-text review of all eligible studies on de-implementation interventions in cancer care delivery. We used the framework analysis approach to summarize included studies’ key characteristics including design, type of cancer, outcome(s), objective(s), de-implementation interventions description, and determinants of the de-implementation interventions. To extract the data, pairs of authors placed text from included articles into the appropriate cells within our framework. We analyzed extracted data from each cell to describe the studies and findings of de-implementation interventions aiming to reduce low-value cancer care.

**Results:**

Out of 2794 studies, 12 met our inclusion criteria. The studies covered several cancer types, including prostate cancer (*n* = 5), gastrointestinal cancer (*n* = 3), lung cancer (*n* = 2), breast cancer (*n* = 2), and hematologic cancers (*n* = 1). Most of the interventions (*n* = 10) were multifaceted. Auditing and providing feedback, having a clinical champion, educating clinicians through developing and disseminating new guidelines, and developing a decision support tool are the common components of the de-implementation interventions. Six of the de-implementation interventions were effective in reducing low-value care, five studies reported mixed results, and one study showed no difference across intervention arms. Eleven studies aimed to de-implement low-value care by changing providers’ behavior, and 1 de-implementation intervention focused on changing the patients’ behavior. Three studies had little risk of bias, five had moderate, and four had a high risk of bias.

**Conclusions:**

This review demonstrated a paucity of evidence in many areas of the de-implementation of low-value care including lack of studies in active de-implementation (i.e., healthcare organizations initiating de-implementation interventions purposefully aimed at reducing low-value care).

**Supplementary Information:**

The online version contains supplementary material available at 10.1186/s13012-022-01197-5.

Contributions to the literature

- We systematically reviewed the literature on the de-implementation of low-value services in cancer care delivery.
- Auditing and providing feedback, having a clinical champion, educating clinicians through developing and disseminating new guidelines, and developing a decision support tool that is often integrated within the electronic health record system are the common components of the de-implementation interventions.
- Our findings highlight the need for moving from passive de-implementation (i.e., clinicians voluntarily follow the new guidelines and decide to change the way they practice) to active de-implementation (i.e., organizations initiating interventions aimed at reducing the low-value care).

## Background

The National Cancer Institute estimates that the cost of cancer-related medical services and prescription drugs will be over $246 billion by 2030 [1]. One method of controlling cancer care costs without reducing the quality of care is to de-implement low-value services. While there is no universally accepted definition of de-implementation, it is generally defined as reducing, replacing, or stopping (partially or completely) low-value services [2, 3]. The National Academy of Medicine defines a low-value service as one where the potential risk of harm outweighs the potential benefits, wastes patients’ time or money, and does not increase the value of care to the patient [4, 5]. For example, prostate-specific antigen (PSA) screening for average-risk men [6, 7], lung cancer screening for asymptomatic patients [8], and axillary staging and post-lumpectomy radiotherapy in women older than 70 years of age with clinically node-negative, hormone receptor + breast cancer [9] are considered low-value services in cancer care delivery. Given that there are known low-value services in cancer care [10, 11], this presents an important setting to systematically evaluate de-implementation efforts.

Medical societies have developed several guidelines and recommendations pertaining to low-value tests, treatments, and follow-up processes across the cancer care continuum [12–14]. However, recent reviews found that a considerable proportion of services that cancer patients receive could still be classified as low-value [15–17]. For example, both the American Society for Clinical Oncology and Choosing Wisely Canada [18, 19] recommend not to use imaging in early-stage breast cancer; despite these recommendations, about one-third of early-stage breast cancer patients underwent at least one advanced imaging exam (e.g., bone scan, positron-emission tomography) for staging [20, 21]. While studies were conducted to explore why so few low-value clinical practices are de-implemented [22, 23], available studies have primarily focused on changes in clinicians’ practice patterns over time in response to educational campaigns (e.g., Choosing Wisely [13]), guidelines (e.g., European Society of Medical Oncology guidelines), or dissemination of scientific publications. Additionally, while the impact of patient-level factors on interventions’ sustainability is well studied [24], little is known about how patient-level factors (e.g., preferences) may impact de-implementation interventions. Furthermore, our understanding of current de-implementation interventions in cancer care delivery and determinants (i.e., factors that influence outcomes [25]) of effective de-implementation efforts in cancer care is limited. Understanding current de-implementation efforts in cancer care delivery is important because it helps to scale up the use of de-implementation interventions and accelerate the reduction of low-value cancer care.

To our knowledge, there is no systematic review that explores the current landscape of de-implementation of low-value services in cancer care delivery. We conducted this systematic review to summarize the literature on interventions to de-implement low-value care in cancer care delivery. Specifically, we sought to identify the determinants of and assess the effectiveness of de-implementation interventions in cancer care. Findings from this study are expected to inform the literature about opportunities for additional work (i.e., identifying gaps) and define an agenda for future research.

## Methods

We conducted a systematic literature review. We reported the results of the review according to Preferred Reporting Items for Systematic Reviews and Meta-Analyses guidelines (Additional file 1) [26]. The study protocol was registered in the International Prospective Register of Systematic Reviews (PROSPERO) (registration number: CRD42021252482).

### Study inclusion and exclusion criteria

To be included in the review, we required articles to focus on a purposeful effort or intervention to de-implement low-value cancer care. We excluded studies on quality improvement interventions without an active de-implementation component. De-implementation was defined as removing, replacing, reducing, and restricting a low-value service [27]. To identify low-value practices, we used recommendations developed by The American Society of Clinical Oncology and Choosing Wisely Canada [10, 11]. Cancer care delivery was defined as a focus on the diagnosis and treatment of cancer, supportive and survivorship care, and cancer prevention. Additionally, studies were required to be peer-reviewed and report the results of an empirical study. The detailed list of inclusion and exclusion criteria can be found in Table 1.

**Table 1:** Study eligibility criteria

Study characteristic	Inclusion criteria	Exclusion criteria
Population	• Hospitals/clinics • Inpatient units • Outpatient general medical settings (e.g., primary care, urgent care, private offices) • Cancer centers • Emergency departments • Managed care organizations	• Health insurance • Free standing EDs • Nursing home
Intervention	• Interventions that purposefully developed to remove^a^, replace^b^, reduce^c^, restrict^d^, reverse, de-implement, de-adopt, disinvest, decrease in use, discontinue, abandon, reassess, obsolete, withdraw, contradict, refute, delist, substitute, exnovate, cease, or end an established low-value practice	• Changes in clinicians’ practice pattern over time in response to educational campaigns, guidelines, or dissemination of scientific publications without active effort to de-implement an established low value practice • Quality improvement interventions without a de-implementation component
Reasons^e^	• Low value practices^e^ (e.g., ineffective^f^, contradicted^g^, mixed^h^, and untested^i^ interventions)	
Outcome	• De-implementation determinants (i.e., factors influence de-implementation outcomes such as incentives and resources) • De-implementation process (i.e., process of reducing, replacing, or stopping low-value services) • De-implementation outcome (e.g., effectiveness, volume of procedures, cost saving, quality)	• Any outcomes not listed
Study design	• Randomized trials • Quasi-experiment studies • Cross-sectional • Qualitative studies • Case reports and case studies • Interrupted time-series studies or repeated measures studies • Prospective and retrospective observational studies (i.e., cohort studies, case control studies)	• Descriptive studies with no outcomes data • Modeling studies that used simulated data • Not a clinical study (e.g., editorial, nonsystematic review, letter to the editor) • Prospective and retrospective observational studies • Clinical guidelines • Measurement or validation studies • Pilot studies without adequate power to assess impact of intervention on outcomes.
Publication types	• Full publication in a peer-reviewed journal • English-language publications • 1990 to current date	• Non-English language • Not a full publication in a peer-reviewed journal • Letters, editorials, reviews, dissertations, meeting abstracts, protocols without results

^a^Removing an intervention is the process of stopping the delivery of an inappropriate intervention entirely

^b^Replacing an intervention involves stopping an inappropriate intervention and starting a new, evidence-based intervention that targets the same or similar proximal or distal patient-level health behaviors or health outcomes

^c^Reducing an intervention involves changing the frequency and/or intensity with which that intervention is delivered

^d^Restricting an intervention occurs when the scope of an intervention is narrowed by target population, health professional, and/or delivery setting

^e^Low-value practices defined as those identified by by The American Society of Clinical Oncology and Choosing Wisely Canada

^f^Ineffective interventions are those for which a few (if not many) high-quality studies have shown to not improve patients’ health outcomes or behaviors and may actually incur more harm than benefit

^g^Contradicted interventions (i.e., medical reversals) are those for which a newer, higher-quality study (or studies) indicates that the health intervention does not improve outcomes, which is contrast to a previous, lower-quality study (or studies) indicating that it does work

^h^Mixed interventions are those for which the quantity and quality of evidence in support of and against the effectiveness of the intervention is approximately equal

^i^Untested interventions are those for which little to no empirical evidence exists about their effectiveness because they have yet to be studied

### Information sources and search strategy

The literature search strategy was developed by the first author (AA) along with a professional medical research librarian (RC). The search was intentionally broad to minimize the risk of overlooking potentially relevant studies. The search strategy was developed for the concepts of cancer care delivery, low-value care, and de-implementation of cancer-related programs. The search strategies were created using a combination of subject headings and keywords and were used to search PubMed, Embase, CINAHL Plus, and Scopus from 1 January 1990 to 4 March 2021, when all searches were completed. We also manually scanned the citations of included studies for relevant articles in case they were missed during indexing. As we considered only peer-reviewed published studies, gray literature was not included. We applied the Cochrane human studies filter to exclude animal studies and added a systematic review keyword and publication type filter to exclude systematic review articles. The complete strategy for each of the searches can be found in Additional file 2.

### Study selection process

Each title and abstract was screened against the eligibility criteria by two investigators. Discrepancies were resolved through discussions between members of each pair and, when necessary, a third team member reviewed the discrepancy until a consensus was reached. To ensure inter-rater reliability of reviews, three iterations of sample reviews were conducted with each person reviewing 50 articles until an average agreement of 83.38% was reached. The full-text articles were screened in the same manner.

### Study quality assessment

Two independent authors assessed the quality of included studies using three risk of bias tools (based on studies’ methodology) including (1) National Institutes of Health (NIH) Quality Assessment Tool for the controlled intervention studies; (2) NIH Quality Assessment Tool for the before-after (pre–post) studies with no control group studies, and (3) NIH Quality Assessment Tool for the observational cohort and cross-sectional studies [28]. Disagreements in the risk of bias scoring were resolved by consensus or by discussion with a third author.

### Data extraction and analysis

We did not conduct a meta-analysis due to heterogeneity in populations, interventions, and outcomes of the included studies. We used a framework analysis approach to summarize the evidence of de-implementation interventions aiming to reduce low-value cancer care [29]. The framework analysis approach included five stages (i.e., familiarization, framework selection, indexing, charting, and mapping and interpretation.) First, team members read included studies and familiarized themselves with the literature. Second, we identified conceptual frameworks that served as the codes for data abstraction [27, 30, 31]. To describe studies in which researchers have studied de-implementation interventions aiming to reduce low-value cancer care, we used a thematic framework that included publication year, design, outcome(s), type of cancer, objective(s), country, setting, type/name of low-value care, de-implementation intervention description (e.g., type, name), single or multifaceted de-implementation intervention strategy, any framework/conceptual or theoretical model used, barriers to de-implementation intervention use, the effectiveness of the de-implementation intervention, and assessment of patients’ priorities/perceptions when de-implementing the low-value care. Next, pairs of authors completed indexing and charting by placing selected text from included articles into the appropriate cells within our framework. Data from the included studies were extracted into a standardized data extraction form in Microsoft Excel (version 2016). Last, we analyzed extracted data from each cell to describe the studies and findings of de-implementation interventions aiming to reduce low-value cancer care.

## Results

### Study selection

**Fig. 1:**
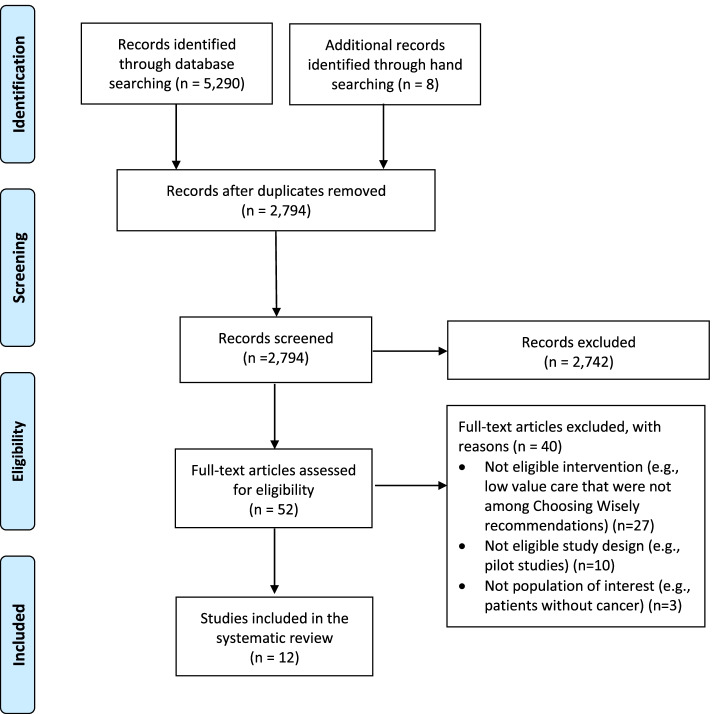
PRISMA Literature Flow Diagram

The searches in PubMed, Embase, CINAHL, and Scopus yielded 5290 citations. These citations were exported to Endnote (Version 20) and 2504 duplicates were removed using the Endnote deduplication feature. Additionally, eight records were identified through hand searching. This resulted in a total of 2794 unique citations found across all database searches. Titles and abstracts of the 2794 articles were screened; 52 were selected for full-text screening. Of the 52 studies, 40 were excluded at full-text screening or during extraction attempts with the consensus of two coauthors; 12 unique eligible studies were included [32–43] (Fig. 1).

### Characteristics of included studies

The included studies were published between 2003 and 2020. Most included studies (*n* = 10) used either interrupted time series or pre–post-study designs [33–35, 37–43], one study was an observational cohort study [36], and one study was randomized clinical trials [32]. Most of the included studies (*n* = 8) were conducted in the USA [32, 33, 35, 36, 39, 40, 42, 43]. The remaining studies were conducted in England (*n* = 2) [37, 41], France (*n* = 1) [38], and Netherlands (*n* = 1) [34]. The studies covered several cancer types including prostate cancer (*n* = 5) [32, 35, 39, 40, 43], gastrointestinal cancer (*n* = 3) [32, 36, 38], lung cancer (*n* = 2) [36, 42], breast cancer (*n* = 2) [33, 36], and hematologic cancers (*n* = 1) [37]. Some of the included studies focused on more than one cancer type [32, 34, 36, 41]. Five studies focused on low-value screening services (e.g., inappropriate PSA-based prostate cancer screening among men aged 75 and over) [32, 35, 39, 40, 43], two studies focused on de-implementing low-value diagnostic tests (e.g., ordering of diagnostic markers) [33, 38], and five studies focused on de-implementing low-value treatment procedures (e.g., inappropriate use of peripheral intravenous and urinary catheters) [34, 36, 37, 41, 42]. Characteristics of included studies are shown in Table 2.

**Table 2:** Characteristic of included studies

Citation	Design	Country	Setting	Participants	Outcomes	Low-value care	Study primary objective
**Durieux et al. (2003)** [38]	Interrupted time series	France	An academic medical center	Patients with GI tumors	The number of tumor markers ordered by physicians and the number of admissions	Inappropriate ordering of three tumor markers (carcinoembryonic antigen, alpha-fetoprotein, carbohydrate antigen 19-9)	To evaluate the long-term impact of an intervention designed to reduce the ordering of three tumor markers frequently prescribed for gastroenterological diseases (carcinoembryonic antigen, alpha-fetoprotein, carbohydrate antigen 19-9).
**Miller et al. (2011)** [43]	Before and after study	USA	Multi-site urology practices	Patients with prostate cancer	Use of bone scans and computerized tomography across prostate cancer risk strata	Imaging in patients with low-risk prostate cancer	To describe findings from a Urological Surgery Quality Collaborative project focused on improving the use of radiographic staging in men with newly diagnosed prostate cancer.
**Butler et al. (2015)** [37]	Before and after study	UK	An academic medical center	Patients with hematologic cancers	The proportion of noncompliant transfusions received above the recommended triggers. The total number of RBCs and PLTs received during the study period, proportion of patients transfused, mean pretransfusion Hb level and PLT count, mean post-transfusion Hb level and PLT count, and time delay between pre- and post-transfusion full blood count and the receipt of blood products.	Unnecessary blood transfusion	To assess the impact of a clinical decision support system for blood product ordering in patients with hematologic disease.
**Ross et al. (2015)** [39]	Before and after study	USA	Urology practices	Patients with prostate cancer	The number of bone scan and CT scans	Imaging in patients with low-risk prostate cancer	To determine whether collaborative-wide data review and performance feedback would decrease the imaging rate in men with low-risk prostate cancer.
**Shelton et al. (2015)** [40]	Interrupted time series	USA	Outpatient clinics, academic and ambulatory care centers (VA Medical Centers)	Patients with prostate cancer	Monthly PSA-based prostate cancer screening rate in unique patients who had a visit to any primary care clinic.	PSA-based screening for prostate cancer in men aged 75 years and older	To determine whether a highly specific computerized clinical decision support alert to remind providers, at the moment of PSA screening order entry, of the current guidelines and institutional policy would reduce the use of inappropriate PSA-based prostate cancer screening among men aged 75 and over.
**Martin Goodman et al. (2016)** [42]	Before and after study	USA	A comprehensive cancer center	Patients with non–small-cell lung cancer	Patients with non–small-cell lung cancer who received pegylated granulocyte colony-stimulating factor (pGCSF) for low- or intermediate-risk febrile neutropenia chemotherapy regimens	Inappropriate use of prophylactic pegylated granulocyte colony-stimulating factor in patients with less than 10% risk of neutropenic fever	To examine the baseline rate of primary prophylactic pGCSF administration for patients with non–small-cell lung cancer, increase provider awareness of appropriate pGCSF use, and minimize the prescription of primary prophylactic pGCSF for patients with lung cancer who are treated with low-risk chemotherapy regimens, without a negative impact on patient safety.
**Sheridan et al. (2016)** [32]	Randomized clinical trial	USA	Community-based practices	Patients with prostate or colorectal cancer	The change in intention to accept screening. General and disease-specific knowledge, perceived risk and consequences of disease, screening attitudes, perceived net benefit of screening, values clarity, and self-efficacy for screening.	Prostate cancer screening in men ages 50–69 years and colorectal cancer screening in men and women ages 76–85 years	To examine the comparative effectiveness of 4 alternate formats for presenting benefits and harms information in reducing intentions for screening and changing secondary behavioral and decision-making outcomes for patients eligible for 1 of 3 low-value or potentially low-value screening services.
**Hill et al. (2018)** [33]	Before and after study	USA	A comprehensive interdisciplinary breast center	Patients with breast cancer	Frequency of ordering CBC and LFTs (overall and per provider), subsequent testing prompted by abnormal results, and overall compliance with guidelines.	Ordering complete blood cell count and liver function tests in patients with early breast cancer	To measure compliance with guidelines for ordering complete blood cell count (CBC) and liver function tests (LFT) before and after the calendar date when the guidelines transitioned from routine to unnecessary.
**Gob et al. (2019)** [41]	Before and after study	UK	A tertiary care unit	Patients admitted to oncology unit	The percentage of one-unit red cell transfusion orders (aggregated monthly).	Two-unit red cell transfusion orders.	Assess the proportion of one-unit red cell transfusion orders on the oncology ward
**Hoque et al. (2020)** [36]	Observational cohort study	USA	VA Medical Centers	Patients with colorectal, breast and non-small cell lung cancer	Erythropoisis stimulating agent treatment use and transfusion, and venous thromboembolism occurrence and mortality	ESA treatment use and transfusion	Evaluate the influence of FDA black box warnings and risk evaluation monitoring strategies on use of erythropoiesis stimulating agent in Veterans Administration cancer patients with chemotherapy induced anemia.
**Ciprut et al. (2020)** [35]	Before and after study	USA	VA Medical Center	Patients with prostate cancer	Effectiveness (number of imaging) and acceptability of an EMR-based Clinical Reminder Order Check intervention	Imaging in patients with low-risk prostate cancer	To understand how to potentially improve inappropriate prostate cancer imaging rates.
**Laan et al. (2020)** [34]	Interrupted time series	Holland	University and general hospitals	Patients admitted to oncology unit	Percentages of short peripheral intravenous catheters, catheter-related infections and other complications, catheter reinsertion rate, use of antibiotics, hospital length of stay (and ICU), and mortality	Inappropriate use of peripheral intravenous and urinary catheters	To reduce inappropriate use of catheters to reduce health care-associated infections.

### Quality assessment of studies

The overall quality of an included randomized clinical trial was good (assessed by NIH Quality Assessment Tool for the controlled intervention studies) [32]. The overall quality of an included observational cohort study was fair (assessed by NIH Quality Assessment Tool for the observational cohort and cross-sectional studies) [36]. The overall quality of four of the pre–post designs studies was poor [33, 39, 41, 43], the quality of four of them was fair [35, 37, 38, 42], and the quality of two of them was good (assessed by NIH Quality Assessment Tool for the before-after (pre–post) studies with no control group studies) [34, 40]. The details of the quality assessment of the included studies are shown in Additional file 3.

### De-implementation interventions’ characteristics

All included studies described at least one de-implementation intervention. De-implementation interventions’ characteristics can be found in Table 3. From the four types of de-implementation action (i.e., removing, replacing, reducing, and restricting) [27], all of the actions in included studies aimed at reducing low-value care without offering a high-value care replacement. Most of the implemented interventions (*n* = 11) were multifaceted (i.e., interventions included two or more components) [33–43]. Developing a decision support tool (*n* = 11) (usually integrated within the electronic health record system to assist clinicians) [32–38, 40–43], auditing and providing feedback (*n* = 7) [33, 34, 37, 39, 41–43], educating clinicians through developing and disseminating new guidelines (*n* = 5) [33, 34, 41–43], and having a clinical champion (*n* = 3) [34, 39, 43] are the common components of many of the de-implementation interventions. Only one of the de-implementation interventions (i.e., one-page, written evidence-based decision support sheet to present benefits and harms information in reducing intentions for screening) focused on changing the patients’ behavior [32]. Other studies aimed at de-implementing low-value care by changing providers’ behavior.

**Table 3:** Characteristics of interventions implemented to de-implement low-value care in cancer care delivery

Citation	De-implementation intervention description	The effects of the de-implementation intervention	Determinants of the use of the de-implementation intervention
**Durieux et al. (2003)** [38]	A specific laboratory order form with clinical guidelines to improve appropriate test orders.	The number of tumor markers prescribed, and the ratio tumor markers/admissions decreased in the hospital (*p* < .0001), and in the Departments of Gastroenterology (*p* < .0001) and Internal Medicine (*p* < .01).	Local adaptation of guidelines by those who are going to use them, implementation strategy for guidelines, and scientific knowledge concerning the utility of different markers.
**Miller et al. (2011)** [43]	A multistep intervention including (1) audit and comparative performance feedback, (2) having a clinical champion, (3) dissemination of clinical guidelines, and (4) establishing the Urological Surgery Quality Collaborative as an infrastructure for physician led, collaborative quality improvement in urology.	Compared with baseline practice patterns (31% bone scans, 28% computerized tomography), urologists in Urological Surgery Quality Collaborative practices ordered fewer bone and computerized tomography scans in post-intervention phases 2 (23%, 21%) and 3 (16%, 13%) of data collection (*p* < 0.01), including a significant reduction in the use of these studies in patients with low and intermediate risk cancer (*p* < 0.05).	Not reported
**Butler et al. (2015)** [37]	Computerized physician order entry systems have been integrated with a clinical decision support system software to improve compliance with restrictive blood management protocols. Such systems require physicians to specify the indication for blood product transfusion and highlight to the clinician the requests that lie outside prespecified guidelines for transfusion by linking them to the most recent laboratory results. In addition, extensive, real-time education, support, and feedback were provided to clinicians.	There was no significant difference in (1) the mean number of transfusions per patient, (2) the proportion of patients transfused, (3) post-transfusion hemoglobin (Hb), and (4) pre- and post-transfusion PLT count, although mean pretransfusion Hb decreased. The proportion of noncompliant RBC and PLT transfusion requests improved from baseline to CDSS2 (69.0 to 43.4% *p* < 0.005 for RBCs, and 41.9 to 31.2%, *p* = 0.16 for PLT).	The amount of time and human resources required to provide monitoring, analysis, and feedback, and provider reluctance.
**Ross et al. (2015)** [39]	A multistep intervention including (1) audit and performance feedback, (2) having a clinical champion.	Bone scan decreased from 3.7 to 1.3% (*p* = 0.03), and computerized tomography decreased from 5.2 to 3.2% (*p* = 0.17).	Not reported
**Shelton et al. (2015)** [40]	A clinical computerized decision support (CCDS) tool to remind providers of current recommendations against PSA-based prostate cancer screening for men 75 and older. A pop-up message to alert providers ordering a screening PSA test in a patient 75 years of age or older. When triggered, a brief interruptive educational message was shown on the ordering screen.	The mean monthly screening rate decreased from 8.3 to 4.6%. The screening rate declined by 38% during the baseline period and by 40% and 30%, respectively, during the two periods when the CCDS tool was turned on. The screening rate ratios for the baseline and two periods when the CCDS tool was on were 0.97, 0.78, and 0.90, respectively, with a significant difference between baseline and the first CCDS-on period (*p* < 0.0001), and a trend toward a difference between baseline and the second CCDS-on period (*p* = 0.056).	The alert fatigue, difficulty in changing providers’ behavior, and the rotation of resident physician staff
**Martin Goodman et al. (2016)** [42]	Three Plan-Do-Study-Act cycles, educated providers about the appropriate use and cost of pGCSF, developed the Cleveland Clinic consensus guidelines, removed primary prophylactic pGCSF from LRCR EMR orders.	The percentage of patients who received inappropriate primary prophylactic pGCSF and the number of doses per patient decreased significantly. Cost analysis showed an average 86% decrease in billed charges per month, which would result in $408,000 in annual savings based on the current CMS allowable payment per dose.	Not reported
**Sheridan et al. (2016)** [32]	One-page, written evidence-based decision support sheet.	Intentions to accept screening were high before the intervention and change in intentions did not differ across intervention arms (words, − 0.07; numbers, − 0.05; numbers plus narrative, − 0.12; numbers plus framed presentation, − 0.02; *P* = .57 for all comparisons). Change in other outcomes also showed no difference across intervention arms.	Not reported
**Hill et al. (2018)** [33]	A planned implementation strategy using levels of the National Quality Strategy including (1) learning and technical assistance, (2) measurement and feedback, (3) certification, accreditation, and regulation, (4) innovation and diffusion (of quality improvement strategies), (5) workforce development, (6) patient education, (7) reward providers, and (8) modify the existing electronic medical record synoptic documentation template.	The overall rate of compliance with guidelines for ordering a CBC and LFT was 82% and 87%, respectively. Segregated by the pre- and post-guideline change time period, the compliance rates for ordering a CBC and LFT were 78% and 87% (*P* = 0.076).	(1) Integrated health care systems, (2) resource availability (e.g., electronic medical records and funding for academic research assistants)
**Gob et al. (2019)** [41]	A multistep intervention including (1) a root cause analysis targeted at discovering contributing factors to two-unit transfusion orders, including a retrospective audit of the previous month’s two-unit transfusions, structured brain-storming by the study authors, and focused interviews with house staff and attending physicians. (2) An educational campaign with an educational email, and a Grand Rounds presentation focusing on improving awareness of the Choosing Wisely guidelines. (3) A real-time audit and feedback, (4) focused oncologist interviews, (5) modify the transfusion orders setting.	Modifying the transfusion orders templates was the only intervention that resulted in an immediate and sustained change to the system. Post-intervention, the mean proportion of one-unit transfusions rose to 86.0% and was sustained for the 17 months of ongoing data collection.	Bias inertia toward low-value care (i.e., status quo bias)
**Hoque et al. (2020)** [36]	FDA black box warnings and risk evaluation monitoring strategies	ESA use for epoetin fell from 22 to 1%, and for darbepoetin fell from 11 to 1% (*p* < 0.01). Mean hematocrit levels at ESA initiation decreased from 30 to 21% (*p* < 0.01).	National policies and regulatory decisions, patient consent
**Ciprut et al. (2020)** [35]	Using the National Comprehensive Cancer Network’s guidelines, a Clinical Reminder Order Check (CROC) that alerts ordering providers of potentially inappropriate imaging orders in real-time based on patient features of men diagnosed with low-risk prostate cancer	The percentage of the men who were staged according to guidelines increased from 65 to 81%. Inappropriate imaging of men with low-risk prostate cancer was reduced by 16%.	Not reported
**Laan et al. (2020)** [34]	A tailored multi- faceted intervention incudes an assessment of determinants of practice for inappropriate catheter use nurse education, physician champion, empowerment of nurses depending on the local situation of the participating hospital, audit and feedback, and additional interventions such as smart phrase for the daily patient report in electronic health records.	Inappropriate use of peripheral intravenous catheters decreased from 22.0 to 14.4% (incidence rate ratio [IRR] 0.65, 95% CI 0·56 to 0.77, *p* < 0·0001). An absolute reduction in inappropriate use of peripheral intravenous catheters from baseline to intervention periods of 6.65% (95% CI 2.47 to 10.82, *p* = 0·011). Inappropriate use of urinary catheters decreased from 32.4 to 24.1% (IRR 0.74, 95% CI 0.56 to 0.98, *p* = 0·013). An absolute reduction in inappropriate use of urinary catheters of 6.34% (95% CI – 12.46 to 25.13, *p* = 0·524).	Piloting the intervention, developing evidence-based and consensus-driven criteria for appropriate use of peripheral intravenous catheters

### De-implementation interventions’ determinants

While none of the included studies systematically assessed the determinants of de-implementing interventions, six of them mentioned some of the determinants such as clinicians’ lack of confidence and trust in the new evidence, bias inertia toward low-value care (i.e., status quo bias), and resource availability [33, 34, 36–38, 40, 41]. None of the included studies applied de-implementation theories, models, and frameworks to identify the determinants of the use of the de-implementation interventions. However, all the included studies developed their de-implementation interventions based on behavioral, communication, and economic theories or published guidelines (e.g., National Comprehensive Cancer Network guidelines for staging evaluations in men with early-stage prostate cancer).

### De-implementation interventions’ effectiveness

The main objective of all included studies was to test the effectiveness of de-implementation interventions (e.g., examining the effectiveness of alternate formats for presenting benefits and harms information in reducing intentions for unnecessary prostate and colorectal cancer screening). Six of the de-implementation interventions were effective in reducing low-value care [35, 36, 38, 40, 42, 43], five studies reported mixed results (e.g., a multistep intervention including collaborative-wide data review and performance feedback significantly decreased the bone scan rate from 3.7 to 1.3% (*P* = 0.03), while it decreased computerized tomography from 5.2 to 3.2% in a non-significant way (*P* = 0.17)) [33, 34, 37, 39, 41], and in one study outcomes showed no difference across intervention arms [32]. The most effective component among interventions was integrating a decision support tool (e.g., a clinical computerized decision support tool that alerts clinicians of potentially inappropriate orders in real-time) within the electronic health record system [35, 36, 38, 40–43]. Gob et al. study showed among many interventions they have implemented to increase the proportion of one-unit red cell transfusion orders (vs. two units), modifying the transfusion orders templates was the only intervention that resulted in an immediate and sustained change to the system [41].

## Discussion

This systematic review aimed to summarize existing de-implementation efforts in cancer care delivery, including what types of interventions were developed, their effectiveness, and factors that may affect the use of the de-implementation intervention. We found most of the studies were published in recent years (i.e., after 2015) and were conducted in the USA. We found that majority of de-implementation interventions were multi-faceted, and they were successful in reducing low-value care. Included studies covered over-utilization across the cancer care continuum (i.e., over-screening, over-diagnosis, and over-treatment) with a focus on over-screening (e.g., for prostate cancer). Most of the de-implementation interventions structured as multistep interventions followed the Plan-Do-Study-Act cycle. Generally, first, a multidisciplinary team led by a clinical champion audited the clinicians’ practice data, compared the data with evidence-based guidelines (e.g., National Comprehensive Cancer Network guidelines). Second, the baseline practice data and discrepancies with evidenced-based guidelines are presented to clinicians at each practice (i.e., feedback). This is an important step as a recent review finds providing feedback to clinicians is associated with reducing overuse of tests and treatments, and increasing guidelines adherence [44]. Additionally, providing clinicians with evidence-based interventions through educational programs can help them to substitute low-value care with high-value care. A systematic review on the effects of de-implementation interventions aimed at reducing low-value nursing procedures showed the majority of the studies with a positive significant effect used a de-implementation strategy with an educational component [45]. Many of included studies also mentioned developing a clinical decision support tool often integrated within the electronic health record system (e.g., a pop-up message to alert clinicians) to assist clinicians to reduce low-value care. Different studies showed guideline enforcement strategies (e.g., clinical decision support tools) are the most effective strategies to reduce low-value services [41, 46]. Our review also revealed a paucity of evidence in five key areas.

First, we found that very few interventions have been used to de-implement low-value cancer care practices. Lack of de-implementation interventions to reduce low-value care may explain why low-value cancer care persists, despite significant forces over the past decade to reduce low-value care [13, 14]. While educational campaigns and medical guidelines have shown some potential in raising awareness regarding low-value services in cancer care [47], recent studies demonstrate that, in many areas, those recommendations had a limited effect on reducing low-value care [48, 49]. For example, Encinosa et al. found while the odds of antiemetic overuse decreased significantly during the first 6 months after the dissemination of Choosing Wisely recommendations, the decrease however was temporary, and it increased again after 6 months [49]. De-implementation is a planned process that involves interaction between multilevel and multifaceted factors [50]. Therefore, simply diffusing evidence without active efforts to abandon a particular low-value practice is unlikely to lead to meaningful results. This finding highlights the need for moving from passive de-implementation (i.e., solely relying on disseminating evidence and expecting that clinicians will voluntarily follow new guidelines) to active de-implementation (i.e., implementing interventions purposefully aimed at reducing low-value care, such as workflow modification and systems facilitating change).

Second, the focus of all the included studies was to test the effectiveness of de-implementation interventions. Future studies should focus on other aspects of de-implementation interventions such as the relationship between de-implementation interventions and health disparities, and the unintended consequences of the de-implementing of an intervention [27]. Studying the relationship between de-implementation interventions and health disparities is needed to ensure that de-implementation efforts do not exacerbate existing inequities. Not all populations react to de-implementation efforts in the same way [51, 52]. For example, while prior research showed Black and Hispanic Americans are at higher risk of both overuse of low-value care and underuse of high-value care [53], they have been found to perceive de-implementation efforts as withholding potentially beneficial care [54]. Additionally, none of the included studies assessed the unintended consequences of the de-implementation interventions. This is an important gap because de-implementation interventions may have unintended consequences that affect patients, such as increased distrust of the health care system, questioning of underlying motives of de-implementation (e.g., patients may perceive de-implementation interventions as cost-cutting efforts), and undermining patient autonomy [55–57]. It is therefore imperative that the future evaluations of de-implementation interventions consider broader measures to assess the de-implementation interventions’ effects, both positive and negative.

Third, medical centers in included studies developed their de-implementation interventions based on behavioral, communication, and economic theories or published guidelines. However, none of the included studies used de-implementation theories, models, or frameworks to inform their conceptualization or to identify the determinants of using de-implementation interventions. Determinants of implementation and de-implementation may have many similarities (e.g., they both need leadership engagement); however, some elements may be unique to de-implementation [58]. For example, Helfrich et al. highlighted that de-implementation may require a process of unlearning to change knowledge, intentions, and beliefs about a low-value service [46]. Recent reviews identified some theories, models, or frameworks specifically developed for the de-implementation of low-value care [59, 60]. Using theoretical frameworks specifically developed for de-implementation of low-value care therefore may help researchers to better evaluate the de-implementation process, identify determinants of de-implementing low-valued care, and explore interactions among de-implementation determinants (e.g., peer pressure may moderate the influence of guidelines) [35].

Fourth, we found only a few of included studies mentioned the determinants of de-implementation of low-value care practices. This is an important gap, because similar to implementing a novel intervention or policy, de-implementation of a low-value practice is a complex process that is influenced by multi-level factors (i.e., individual-level and organizational level factors) [27, 61, 62]. Many individual-level factors (patient- and clinician-level) may contribute to the success of de-implementation interventions [63]. For example, patients’ perspectives and preferences [45], and trust in their clinicians [64] are key determinants of many practices in cancer care, and ignoring those factors in efforts to de-implement low-value care may jeopardize the de-implementation process. Prior research also showed that most patients overestimate the benefits and underestimate the harms of medical services [55, 56, 65]. Additionally, recent studies showed clinician-level factors such as knowledge, interpersonal skills, motivation, professional confidence, and beliefs about the consequences of practicing a low value explain why clinicians stop providing certain low-value care while not others [66, 67]. In addition to individual-level factors, many collective-level factors (e.g., organizational culture, leadership, resources, and financial status) also contribute to the utilization of low-value care [45]. In many cases, healthcare organizations intentionally decide to continue providing low-value care. For example, some hospitals, particularly those with financial difficulties, may resist de-implementing low-value practices (e.g., novel experimental technologies) if those services generate significant revenue or provide another relative advantage (e.g., competitive edge) over other hospitals [27, 48, 61]. Future research should consider the association between de-implementation interventions and both individual-level (e.g., perceptions of appropriate use and overuse of health services in oncology) and collective-level factors (e.g., community, organizational characteristics, and reimbursement policies). As previously mentioned, theories, models, and frameworks of determinants of de-implementation may aid in identifying these determinants.

Fifth, almost all the included studies focused on changing clinicians’ behaviors without considering patients’ role. This is an important gap as a systematic review on how low-value breast cancer surgery has been de-implemented in response to Choosing Wisely recommendations identified patient decision-making as a key determinant of de-implementation of low-value breast cancer surgery, suggesting that patients’ role should be included in other de-implementation studies [68]. Many studies showed involving patients in deciding a course of care (i.e., shared decision-making) is a powerful tool for reducing low-value care [22, 62, 69, 70]. However, none of the included studies used a shared decision-making process between the clinician and the patient regarding the use of a specific potentially low-value service. Besides, patients’ attitudes toward different parts of the cancer care continuum are different. For example, while patients are generally in favor of taking fewer medications, they also believe that more testing and screening lead to better outcomes [55]. Research is needed to explore the relationship between different categories of low-value cancer care (e.g., screening, testing, treatment) and de-implementation intervention effectiveness.

This review has some limitations. First, we limited our systematic reviews to English-only articles which could result in biased estimates of effect and reduce generalizability. Second, many practices such as inappropriate use of antibiotics to manage febrile neutropenia (e.g., administering empiric vancomycin) could be considered as low-value care [71]. However, we did not include studies on discontinuing such practices (e.g., antibiotic stewardship interventions) because they were not among Choosing Wisely recommendations. Finally, because of challenges in identifying low-value health care [72], it is possible that some organizations de-implemented interventions without labeling them as low-value care. Therefore, despite a comprehensive literature search, there remains a possibility that we may have missed relevant studies. Additionally, all the included studies were conducted in the USA or European countries; therefore, the findings may not be generalizable to other regions.

## Conclusion

This review demonstrated a paucity of evidence in many key areas of the de-implementation of low-value care in cancer care delivery. First, the assumption that new evidence, guidelines, and reimbursement policies alone will change the way clinicians practice is likely misplaced. Relying on clinicians to change their practice in the absence of well-designed de-implementation interventions is unlikely to reduce low-value care. Second, future research should include a broader range of variables when studying de-implementation. Factors such as patients’ perspectives and preferences; patient satisfaction; and system-level factors such as organizational culture, leadership, and resources are understudied yet likely relevant de-implementation determinants. Finally, future studies should assess unintended effects of de-implementing low-value care, such as increased distrust of the health care system, and undermining patient autonomy.

## Supplementary Information

**Additional file 1.** PRISMA 2020 Checklist.

**Additional file 2.** De-implementing Low-Value Care in Cancer Care Delivery Search Strategy.

**Additional file 3.** Quality Assessment Tools Used for Assessing the Quality of included Studies.

## Data Availability

The data used and/or analyzed during the current study are available from the corresponding author on reasonable request and subject to IRB guidelines.

## Abbreviations

NIH: National Institutes of Health
PRISMA: Preferred Reporting Items for Systematic Reviews and Meta-analyses
PSA: Prostate-specific antigen
