# Supplementary material for: De-implementing low-value care in cancer care delivery: a systematic review
Source: Implement Sci. 2022 Mar 12;17:24. doi: 10.1186/s13012-022-01197-5 (PMC8917720; doi:10.1186/s13012-022-01197-5)
Supplement: Supplementary file 2 — Additional file 2. De-implementing Low-Value Care in Cancer Care Delivery Search Strategy. [file 13012_2022_1197_MOESM2_ESM.docx]

**De-implementing Low-Value Care in Cancer Care Delivery Search Strategy**

All studies before duplicate removal: 5,290

All studies after duplicate removal: 2,786

**PubMed Search – Run 03/04/2021**

| Search | Query | Items Found |
| --- | --- | --- |
| 1 | (Neoplasms[mesh] OR neoplasms[tiab] OR neoplasm[tiab] OR neoplasia[tiab] OR neoplasias[tiab] OR neoplastic[tiab] OR dysplastic[tiab] OR dysplasia[tiab] OR dysplasias[tiab] OR "Early Detection of Cancer"[Mesh] OR cancer[tiab] OR cancers[tiab] OR cancerous[tiab] OR malignant[tiab] OR malignancy[tiab] OR malignancies[tiab] OR metastatic[tiab] OR metastasis[tiab] OR metastases[tiab] OR "Biomarkers, Tumor"[Mesh] OR tumor[tiab] OR tumors[tiab] OR tumour[tiab] OR tumours[tiab] OR adenocarcinoma[tiab] OR adenocarcinomas[tiab] OR carcinoma[tiab] OR carcinomas[tiab] OR sarcoma[tiab] OR sarcomas[tiab] OR lymphoma[tiab] OR lymphomas[tiab] OR melanoma[tiab] OR melanomas[tiab] OR leukemia[tiab] OR leukemias[tiab] OR "Cancer Care Facilities"[Mesh] OR "Oncology Service, Hospital"[Mesh] OR oncology[tiab] OR oncologic[tiab] OR chemotherapy[tiab] OR chemotherapies[tiab] OR neoadjuvant therapy[tiab] OR neoadjuvant therapies[tiab] OR chemoradiotherapy[tiab] OR chemoradiotherapies[tiab] OR radioimmunotherapy[tiab] OR radiotherapy[tiab] OR radioimmunotherapies[tiab]) | 4,709,592 |
| 2 | (de-implement[tiab] OR de-implementation[tiab] OR de-implementing[tiab] OR deimplement[tiab] OR deimplementation[tiab] OR deimplementing[tiab] OR deprescriptions[tiab] OR de-adopt[tiab] OR de-adoption[tiab] OR de-adopting[tiab] OR deadopt[tiab] OR deadoption[tiab] OR deadopting[tiab]) | 284 |
| 3 | (decrease-use[tiab] OR decreasing-use[tiab] OR discontinue[tiab] OR discontinuation[tiab] OR de-list[tiab] OR de-listing[tiab] OR delist[tiab] OR delisting[tiab] OR dis-invest[tiab] OR disinvest[tiab] OR disinvestment[tiab] OR de-commission[tiab] OR decommission[tiab] OR de-fund[tiab] OR ex-novate[tiab] OR ex-novation[tiab] OR ex-novating[tiab] OR exnovate[tiab] OR exnovation[tiab] OR exnovating[tiab] OR re-assess[tiab] OR reassess[tiab] OR re-assessing[tiab] OR re-assessment[tiab] OR withdraw[ti] OR withdrawn[tiab] OR contradict[tiab] OR contradicting[tiab] OR refute[tiab] OR refutation[tiab] OR refuting[tiab] OR substitution[tiab] OR restrict[tiab] OR restriction[tiab] OR restricting[tiab] OR abandon[tiab] OR abandoning[tiab] OR ceased[tiab] OR ceasing[tiab] OR ending[tiab] OR ended[tiab]) AND (guideline[tiab] OR guidelines[tiab] OR program[tiab] OR programs[tiab] OR policy[tiab] OR policies[tiab] OR intervention[tiab] OR interventions[tiab] OR health practice[tiab] OR health practices[tiab] OR health-care practice[tiab] OR health-care practices[tiab] OR medical practice[tiab] OR medical practices[tiab] OR health intervention[tiab] OR health interventions[tiab] OR health-care intervention[tiab] OR health-care interventions[tiab] OR medical intervention[tiab] OR medical interventions[tiab] OR procedure[tiab] OR procedures[tiab] OR prescription[tiab] OR prescriptions[tiab] OR prescribe[tiab] OR habit[tiab] OR habits[tiab] OR routine[tiab] OR routines[tiab] OR framework[tiab] OR initiative[tiab] OR research[tiab]) | 106,384 |
| 4 | (low value[tiab] OR low-quality[tiab] OR obsolete[tiab] OR untested[tiab] OR contradicted[tiab] OR ineffective[tiab] OR mixed-results[tiab] OR cost-burden[tiab] OR expense[tiab] OR expensive[tiab] OR high-price[tiab] OR budgetary constraints[tiab] OR new-evidence[tiab] OR acceptability[tiab] OR approval[tiab] OR maintenance[tiab] OR appropriateness[tiab] OR appropriate[tiab] OR suitable[tiab] OR suitability[tiab] OR feasible[tiab] OR feasibility[tiab] OR non-achievable[tiab] OR not-achievable[tiab] OR non-advantageous[tiab] OR not-advantageous[tiab] OR non-attainable[tiab] OR not-attainable[tiab] OR practicable[tiab] OR unpractical[tiab] OR impractical[tiab] OR non-viable[tiab] OR workable[tiab] OR non-worthwhile[tiab] OR not-worthwhile[tiab] OR unnecessary[tiab] OR sustainable[tiab] OR sustainability[tiab] OR quality improvement[tiab] OR non-value added[tiab] OR no-value added[tiab] OR productive maintenance[tiab] OR over-use[tiab] OR over-used[tiab] OR over-diagnosis[tiab] OR over-diagnoses[tiab]) | 1,907,271 |
| 5 | #2 OR #3 | 106,611 |
| 6 | #1 AND #4 AND #5 | 2,101 |
| 7 | #6 AND English[language] AND ("2000"[Date - Publication] : "3000"[Date - Publication]) | 1,709 |
| 8 | #7 NOT ((review[Publication Type] OR guideline[Publication Type] OR practice guideline[Publication Type] OR literature-review[ti] OR systematic review[pt] OR meta analysis[pt] OR systematic-review[ti] OR systematic-literature-review[ti] OR scoping-review[ti] OR cochrane-review[ti] OR meta-analysis[ti] OR meta analysis[ti]) OR (systematic[ti] AND review[ti]) OR (Cochrane Database Syst Rev[ta] AND review[pt])) NOT (animals[Mesh] NOT humans[Mesh]) | 1,181 |

PubMed before duplicate removal: 1,181

PubMed after duplicate removal: 1,179

**Embase Search – Run 03/04/2021**

| Search | Query | Items Found |
| --- | --- | --- |
| 1 | ('Neoplasm'/exp OR neoplasms:ti,ab OR neoplasm:ti,ab OR neoplasia:ti,ab OR neoplasias:ti,ab OR neoplastic:ti,ab OR dysplastic:ti,ab OR dysplasia:ti,ab OR dysplasias:ti,ab OR 'Early cancer diagnosis'/exp OR cancer:ti,ab OR cancers:ti,ab OR cancerous:ti,ab OR malignant:ti,ab OR malignancy:ti,ab OR malignancies:ti,ab OR metastatic:ti,ab OR metastasis:ti,ab OR metastases:ti,ab OR 'tumor marker'/exp OR tumor:ti,ab OR tumors:ti,ab OR tumour:ti,ab OR tumours:ti,ab OR adenocarcinoma:ti,ab OR adenocarcinomas:ti,ab OR carcinoma:ti,ab OR carcinomas:ti,ab OR sarcoma:ti,ab OR sarcomas:ti,ab OR lymphoma:ti,ab OR lymphomas:ti,ab OR melanoma:ti,ab OR melanomas:ti,ab OR leukemia:ti,ab OR leukemias:ti,ab OR 'Cancer center'/exp OR oncologic:ti,ab OR chemotherapy:ti,ab OR "neoadjuvant therapy":ti,ab OR chemoradiotherapy:ti,ab OR radioimmunotherapy:ti,ab OR radiotherapy:ti,ab OR "neoadjuvant therapies":ti,ab OR chemoradiotherapies:ti,ab OR radioimmunotherapies:ti,ab OR radiotherapies:ti,ab) | 6,347,568 |
| 2 | (de-implement:ti,ab OR de-implementation:ti,ab OR de-implementing:ti,ab OR deimplement:ti,ab OR deimplementation:ti,ab OR deimplementing:ti,ab OR deprescriptions:ti,ab OR de-adopt:ti,ab OR de-adoption:ti,ab OR de-adopting:ti,ab OR deadopt:ti,ab OR deadoption:ti,ab OR deadopting:ti,ab) | 259 |
| 3 | (decrease-use:ti,ab OR decreasing-use:ti,ab OR discontinue:ti,ab OR discontinuation:ti,ab OR de-list:ti,ab OR de-listing:ti,ab OR delist:ti,ab OR delisting:ti,ab OR dis-invest:ti,ab OR disinvest:ti,ab OR disinvestment:ti,ab OR de-commission:ti,ab OR decommission:ti,ab OR de-fund:ti,ab OR ex-novate:ti,ab OR ex-novation:ti,ab OR ex-novating:ti,ab OR exnovate:ti,ab OR exnovation:ti,ab OR exnovating:ti,ab OR re-assess:ti,ab OR reassess:ti,ab OR re-assessing:ti,ab OR re-assessment:ti,ab OR withdraw:ti,ab OR withdrawn:ti,ab OR contradict:ti,ab OR contradicting:ti,ab OR refute:ti,ab OR refutation:ti,ab OR refuting:ti,ab OR substitution:ti,ab OR restrict:ti,ab OR restriction:ti,ab OR restricting:ti,ab OR ‘abandon’:ti,ab OR ‘abandoning’:ti,ab OR ceased:ti,ab OR ceasing:ti,ab OR ending:ti,ab OR ended:ti,ab) AND (guideline:ti,ab OR guidelines:ti,ab OR program:ti,ab OR programs:ti,ab OR policy:ti,ab OR policies:ti,ab OR intervention:ti,ab OR interventions:ti,ab OR ‘health practice’:ti,ab OR ‘health practices’:ti,ab OR ‘health-care practice’:ti,ab OR ‘health-care practices’:ti,ab OR ‘medical practice’:ti,ab OR ‘medical practices’:ti,ab OR ‘health intervention’:ti,ab OR ‘health interventions’:ti,ab OR ‘health-care intervention’:ti,ab OR ‘health-care interventions’:ti,ab OR ‘medical intervention’:ti,ab OR ‘medical interventions’:ti,ab OR procedure:ti,ab OR procedures:ti,ab OR prescription:ti,ab OR prescriptions:ti,ab OR prescribe:ti,ab OR habit:ti,ab OR habits:ti,ab OR routine:ti,ab OR routines:ti,ab OR framework:ti,ab OR initiative:ti,ab OR research:ti,ab) | 154,712 |
| 4 | ("low value":ti,ab OR low-quality:ti,ab OR untested:ti,ab OR contradicted:ti,ab OR ineffective:ti,ab OR mixed-results:ti,ab OR cost-burden:ti,ab OR expense:ti,ab OR expensive:ti,ab OR high-price:ti,ab OR "budgetary constraints":ti,ab OR new-evidence:ti,ab OR acceptability:ti,ab OR approval:ti,ab OR maintenance:ti,ab OR appropriateness:ti,ab OR appropriate:ti,ab OR suitable:ti,ab OR suitability:ti,ab OR feasible:ti,ab OR feasibility:ti,ab OR non-achievable:ti,ab OR ‘not-achievable’:ti,ab OR non-advantageous:ti,ab OR ‘not-advantageous’:ti,ab OR non-attainable:ti,ab OR ‘not-attainable’:ti,ab OR practicable:ti,ab OR unpractical:ti,ab OR impractical:ti,ab OR non-viable:ti,ab OR workable:ti,ab OR non-worthwhile:ti,ab OR ‘not-worthwhile’:ti,ab OR unnecessary:ti,ab OR sustainable:ti,ab OR sustainability:ti,ab OR "quality improvement":ti,ab OR "non-value added":ti,ab OR "no-value added":ti,ab OR "productive maintenance":ti,ab OR over-use:ti,ab OR over-used:ti,ab OR over-diagnosis:ti,ab OR over-diagnoses:ti,ab) | 2,589,427 |
| 5 | #2 OR #3 | 154,935 |
| 6 | #1 AND #4 AND #5 | 4,713 |
| 7 | #6 AND English:la AND [embase]/lim AND [2000-2021]/py | 4,012 |
| 8 | #7 NOT ('meta analysis'/exp OR 'systematic review'/exp OR systematic-review:ti OR systematic-literature-review:ti OR scoping-review:ti OR cochrane-review:ti OR meta-analysis:ti OR meta analysis:ti OR 'case report'/exp OR case-report:ti OR 'retraction notice'/exp OR 'conference abstract'/it OR protocol:ti) NOT ('animal'/exp NOT 'human'/exp) | 1,431 |

Embase before duplicate removal: 1,431

Embase after duplicate removal: 672

**CINAHL Search – Run 03/04/2021**

| Search | Query | Items Found |
| --- | --- | --- |
| 1 | (MH "Neoplasms+") OR (MH "Oncology") OR (MH "Oncology Care Units") OR (MH "Cancer Patients") OR (MH "Oncologic Care") OR (MH "Chemotherapy, Cancer") OR (neoplasms OR neoplasm OR neoplasia OR neoplasias OR neoplastic OR dysplastic OR dysplasia OR dysplasias OR cancer OR cancers OR cancerous OR malignant OR malignancy OR malignancies OR metastatic OR metastasis OR metastases OR tumor OR tumors OR tumour OR tumours OR adenocarcinoma OR adenocarcinomas OR carcinoma OR carcinomas OR sarcoma OR sarcomas OR lymphoma OR lymphomas OR melanoma OR melanomas OR leukemia OR leukemias OR oncology OR oncologic OR chemotherapy OR chemotherapies OR neoadjuvant therapy OR neoadjuvant therapies OR chemoradiotherapy OR chemoradiotherapies OR radioimmunotherapy OR radiotherapy OR radioimmunotherapies) | 844,946 |
| 2 | (de-implement OR de-implementation OR de-implementing OR deimplement OR deimplementation OR deimplementing OR deprescriptions OR de-adopt OR de-adoption OR de-adopting OR deadopt OR deadoption OR deadopting) | 201 |
| 3 | (decrease-use OR decreasing-use OR discontinue OR discontinuation OR de-list OR de-listing OR delist OR delisting OR dis-invest OR disinvest OR disinvestment OR de-commission OR decommission OR de-fund OR ex-novate OR ex-novation OR ex-novating OR exnovate OR exnovation OR exnovating OR re-assess OR reassess OR re-assessing OR re-assessment OR withdraw OR withdrawn OR contradict OR contradicting OR refute OR refutation OR refuting OR substitution OR restrict OR restriction OR restricting OR abandon OR abandoning OR ceased OR ceasing OR ending OR ended) AND (guideline OR guidelines OR program OR programs OR policy OR policies OR intervention OR interventions OR “health practice” OR “health practices” OR “health-care practice” OR “health-care practices” OR “medical practice” OR “medical practices” OR “health intervention” OR “health interventions” OR “health-care intervention” OR “health-care interventions” OR “medical intervention” OR procedure OR procedures OR prescription OR prescriptions OR prescribe OR habit OR habits OR routine OR routines OR framework OR initiative OR research) | 54,420 |
| 4 | (low value OR low-quality OR obsolete OR untested OR contradicted OR ineffective OR mixed-results OR cost-burden OR expense OR expensive OR high-price OR budgetary constraints OR new-evidence OR acceptability OR approval OR maintenance OR appropriateness OR appropriate OR suitable OR suitability OR feasible OR feasibility OR non-achievable OR not-achievable OR non-advantageous OR not-advantageous OR non-attainable OR not-attainable OR practicable OR unpractical OR impractical OR non-viable OR workable OR non-worthwhile OR not-worthwhile OR unnecessary OR sustainable OR sustainability OR “quality improvement” OR “non-value added” OR “no-value added” OR “productive maintenance” OR over-use OR over-used OR over-diagnosis OR over-diagnoses) | 499,320 |
| 5 | #2 OR #3 | 54,593 |
| 6 | #1 AND #4 AND #5 | 1,083 |
| 7 | #6 AND Limiters - Published Date: 20000101-20211231; English Language; Human | 649 |
| 8 | #7 NOT (TI systematic-review OR TI systematic-literature-review OR TI scoping-review OR TI cochrane-review OR TI meta-analysis OR TI case-report OR TI protocol) | 599 |

CINAHL before duplicate removal: 599

CINAHL after duplicate removal: 265

**Scopus Search – Run 03/04/2021**

| Search | Query | Items Found |
| --- | --- | --- |
| 1 | TITLE-ABS(“neoplasms”) OR TITLE-ABS(“neoplasm”) OR TITLE-ABS(“neoplasia”) OR TITLE-ABS(“neoplasias”) OR TITLE-ABS(“neoplastic”) OR TITLE-ABS(“dysplastic”) OR TITLE-ABS(“dysplasia”) OR TITLE-ABS(“dysplasias”) OR TITLE-ABS(“cancer”) OR TITLE-ABS(“cancers”) OR TITLE-ABS(“cancerous”) OR TITLE-ABS(“malignant”) OR TITLE-ABS(“malignancy”) OR TITLE-ABS(“malignancies”) OR TITLE-ABS(“metastatic”) OR TITLE-ABS(“metastasis”) OR TITLE-ABS(“metastases”) OR TITLE-ABS(“tumor”) OR TITLE-ABS(“tumors”) OR TITLE-ABS(“tumour”) OR TITLE-ABS(“tumours”) OR TITLE-ABS(“adenocarcinoma”) OR TITLE-ABS(“adenocarcinomas”) OR TITLE-ABS(“carcinoma”) OR TITLE-ABS(“carcinomas”) OR TITLE-ABS(“sarcoma”) OR TITLE-ABS(“sarcomas”) OR TITLE-ABS(“lymphoma”) OR TITLE-ABS(“lymphomas”) OR TITLE-ABS(“melanoma”) OR TITLE-ABS(“melanomas”) OR TITLE-ABS(“leukemia”) OR TITLE-ABS(“leukemias”) OR TITLE-ABS(“oncology”) OR TITLE-ABS(“oncologic”) OR TITLE-ABS(“chemotherapy”) OR TITLE-ABS(“chemotherapies”) OR TITLE-ABS(“neoadjuvant therapy”) OR TITLE-ABS(“neoadjuvant therapies”) OR TITLE-ABS(“chemoradiotherapy”) OR TITLE-ABS(“chemoradiotherapies”) OR TITLE-ABS(“radioimmunotherapy”) OR TITLE-ABS(“radiotherapy”) OR TITLE-ABS(“radioimmunotherapies”) | 4,594,772 |
| 2 | TITLE-ABS (“de-implement”) OR TITLE-ABS(“de-implementation”) OR TITLE-ABS(“de-implementing”) OR TITLE-ABS(“deimplement”) OR TITLE-ABS(“deimplementation”) OR TITLE-ABS(“deimplementing”) OR TITLE-ABS(“deprescriptions”) OR TITLE-ABS(“de-adopt”) OR TITLE-ABS(“de-adoption”) OR TITLE-ABS(“de-adopting”) OR TITLE-ABS(“deadopt”) OR TITLE-ABS(“deadoption”) OR TITLE-ABS(“deadopting”) | 356 |
| 3 | (TITLE-ABS(“decrease-use”) OR TITLE-ABS(“decreasing-use”) OR TITLE-ABS(“discontinue”) OR TITLE-ABS(“discontinuation”) OR TITLE-ABS(“de-list”) OR TITLE-ABS(“de-listing”) OR TITLE-ABS(“delist”) OR TITLE-ABS(“delisting”) OR TITLE-ABS(“dis-invest”) OR TITLE-ABS(“disinvest”) OR TITLE-ABS(“disinvestment”) OR TITLE-ABS(“de-commission”) OR TITLE-ABS(“decommission”) OR TITLE-ABS(“de-fund”) OR TITLE-ABS(“ex-novate”) OR TITLE-ABS(“ex-novation”) OR TITLE-ABS(“ex-novating”) OR TITLE-ABS(“exnovate”) OR TITLE-ABS(“exnovation”) OR TITLE-ABS(“exnovating”) OR TITLE-ABS(“re-assess”) OR TITLE-ABS(“reassess”) OR TITLE-ABS(“re-assessing”) OR TITLE-ABS(“re-assessment”) OR TITLE-ABS(“withdraw”) OR TITLE-ABS(“withdrawn”) OR TITLE-ABS(“contradict”) OR TITLE-ABS(“contradicting”) OR TITLE-ABS(“refute”) OR TITLE-ABS(“refutation”) OR TITLE-ABS(“refuting”) OR TITLE-ABS(“substitution”) OR TITLE-ABS(“restrict”) OR TITLE-ABS(“restriction”) OR TITLE-ABS(“restricting”) OR TITLE-ABS(“abandon”) OR TITLE-ABS(“abandoning”) OR TITLE-ABS(“ceased”) OR TITLE-ABS(“ceasing”) OR TITLE-ABS(“ending”) OR TITLE-ABS(“ended”)) AND (TITLE-ABS(“guideline”) OR TITLE-ABS(“guidelines”) OR TITLE-ABS(“program”) OR TITLE-ABS(“programs”) OR TITLE-ABS(“policy”) OR TITLE-ABS(“policies”) OR TITLE-ABS(“intervention”) OR TITLE-ABS(“interventions”) OR TITLE-ABS(“health practice”) OR TITLE-ABS(“health practices”) OR TITLE-ABS(“health-care practice”) OR TITLE-ABS(“health-care practices”) OR TITLE-ABS(“medical practice”) OR TITLE-ABS(“medical practices”) OR TITLE-ABS(“health intervention”) OR TITLE-ABS(“health interventions”) OR TITLE-ABS(“health-care intervention”) OR TITLE-ABS(“health-care interventions”) OR TITLE-ABS(“medical intervention”) OR TITLE-ABS(“medical interventions”) OR TITLE-ABS(“procedure”) OR TITLE-ABS(“procedures”) OR TITLE-ABS(“prescription”) OR TITLE-ABS(“prescriptions “) OR TITLE-ABS(“prescribe”) OR TITLE-ABS(“habit”) OR TITLE-ABS(“habits”) OR TITLE-ABS(“routine”) OR TITLE-ABS(“routines”) OR TITLE-ABS(“framework”) OR TITLE-ABS(“initiative”) OR TITLE-ABS(“research”)) | 290,613 |
| 4 | TITLE-ABS(“low value”) OR TITLE-ABS(“low-quality”) OR TITLE-ABS(“obsolete”) OR TITLE-ABS(“untested”) OR TITLE-ABS(“contradicted”) OR TITLE-ABS(“ineffective”) OR TITLE-ABS(“mixed-results”) OR TITLE-ABS(“cost-burden”) OR TITLE-ABS(“expense”) OR TITLE-ABS(“expensive”) OR TITLE-ABS(“high-price”) OR TITLE-ABS(“budgetary constraints”) OR TITLE-ABS(“new-evidence”) OR TITLE-ABS(“acceptability”) OR TITLE-ABS(“approval”) OR TITLE-ABS(“maintenance”) OR TITLE-ABS(“appropriateness”) OR TITLE-ABS(“appropriate”) OR TITLE-ABS(“suitable”) OR TITLE-ABS(“suitability”) OR TITLE-ABS(“feasible”) OR TITLE-ABS(“feasibility”) OR TITLE-ABS(“non-achievable”) OR TITLE-ABS(“not-achievable”) OR TITLE-ABS(“non-advantageous”) OR TITLE-ABS(“not-advantageous”) OR TITLE-ABS(“non-attainable”) OR TITLE-ABS(“not-attainable”) OR TITLE-ABS(“practicable”) OR TITLE-ABS(“unpractical”) OR TITLE-ABS(“impractical”) OR TITLE-ABS(“non-viable”) OR TITLE-ABS(“workable”) OR TITLE-ABS(“non-worthwhile”) OR TITLE-ABS(“not-worthwhile”) OR TITLE-ABS(“unnecessary”) OR TITLE-ABS(“sustainable”) OR TITLE-ABS(“sustainability”) OR TITLE-ABS(“quality improvement”) OR TITLE-ABS(“non-value added”) OR TITLE-ABS(“no-value added”) OR TITLE-ABS(“productive maintenance”) OR TITLE-ABS(“over-use”) OR TITLE-ABS(“over-used”) OR TITLE-ABS(“over-diagnosis”) OR TITLE-ABS(“over-diagnoses”) | 5,745,816 |
| 5 | #2 OR #3 | 290,938 |
| 6 | #1 AND #4 AND #5 | 2,807 |
| 7 | #6 AND Limiters - Published Year: 2000-2021; English Language | 2,209 |
| 8 | #7 AND NOT (TITLE(“systematic-review”) OR TITLE(“systematic-literature-review”) OR TITLE(“scoping-review”) OR TITLE(“cochrane-review”) OR TITLE(“meta-analysis”) OR TITLE(“case-report”) OR TITLE("protocol")) NOT Document Type: Review | 2,079 |

Scopus before duplicate removal: 2,079

Scopus after duplicate removal: 670
