# Supplementary material for: De-implementing low-value care in cancer care delivery: a systematic review
Source: Implement Sci. 2022 Mar 12;17:24. doi: 10.1186/s13012-022-01197-5 (PMC8917720; doi:10.1186/s13012-022-01197-5)
Supplement: Supplementary file 3 — Additional file 3. Quality Assessment Tools Used for Assessing the Quality of included Studies. [file 13012_2022_1197_MOESM3_ESM.docx]

**Additional file 3-Quality Assessment Tools Used for Assessing the Quality of included Studies**

NIH Quality Assessment Tool for the controlled intervention studies

|  | Sheridan et al. |
| --- | --- |
| Was the study described as randomized, a randomized trial, a randomized clinical trial, or an RCT? | Yes |
| Was the method of randomization adequate (i.e., use of randomly generated assignment)? | Yes |
| Was the treatment allocation concealed (so that assignments could not be predicted)? | Yes |
| Were study participants and providers blinded to treatment group assignment? | Can’t Tell/NA |
| Were the people assessing the outcomes blinded to the participants' group assignments? | Can’t Tell/NA |
| Were the groups similar at baseline on important characteristics that could affect outcomes (e.g., demographics, risk factors, co-morbid conditions)? | Yes |
| Was the overall drop-out rate from the study at endpoint 20% or lower of the number allocated to treatment? | Yes |
| Was the differential drop-out rate (between treatment groups) at endpoint 15 percentage points or lower? | Yes |
| Was there high adherence to the intervention protocols for each treatment group? | Yes |
| Were other interventions avoided or similar in the groups (e.g., similar background treatments)? | Can’t Tell/NA |
| Were outcomes assessed using valid and reliable measures, implemented consistently across all study participants? | Yes |
| Did the authors report that the sample size was sufficiently large to be able to detect a difference in the main outcome between groups with at least 80% power? | Yes |
| Were outcomes reported or subgroups analyzed prespecified (i.e., identified before analyses were conducted)? | Yes |
| Were all randomized participants analyzed in the group to which they were originally assigned, i.e., did they use an intention-to-treat analysis? | Yes |
| **Overall quality rating** | Good |

NIH Quality Assessment Tool for the before-after (pre-post) studies with no control group studies)

|  | Durieux  et al. | Hill  et al. | Ciprut et al. | Gob  et al. | Laan  et al. | Miller  et al. | Ross  et al. | Martin Goodman et al. | Shelton  et al. | Butler  et al. |
| --- | --- | --- | --- | --- | --- | --- | --- | --- | --- | --- |
| Was the study question or objective clearly stated? | Yes | Yes | Yes | Yes | Yes | Yes | Yes | Yes | Yes | Yes |
| Were eligibility/selection criteria for the study population prespecified and clearly described? | No | Yes | Yes | No | Yes | No | No | No | Yes | No |
| Were the participants in the study representative of those who would be eligible for the intervention in the general or clinical population of interest? | Can’t Tell/NA | Yes | Yes | Yes | Yes | Yes | Yes | Yes | Yes | Yes |
| Were all eligible participants that met the prespecified entry criteria enrolled? | Can’t Tell/NA | Can’t Tell/NA | Can’t Tell/NA | Can’t Tell/NA | Can’t Tell/NA | Can’t Tell/NA | Can’t Tell/NA | Can’t Tell/NA | Yes | Can’t Tell/NA |
| Was the sample size sufficiently large to provide confidence in the findings? | Can’t Tell/NA | Can’t Tell/NA | Can’t Tell/NA | Can’t Tell/NA | Can’t Tell/NA | Can’t Tell/NA | Can’t Tell/NA | Can’t Tell/NA | Can’t Tell/NA | Can’t Tell/NA |
| Was the intervention clearly described and delivered consistently across the study population? | Yes | Yes | Yes | Yes | Yes | No | No | Yes | Yes | Yes |
| Were the outcome measures prespecified, clearly defined, valid, reliable, and assessed consistently across all study participants? | Yes | No | Yes | Yes | Yes | No | No | Yes | Yes | Yes |
| Were the people assessing the outcomes blinded to the participants' exposures/interventions? | Can’t Tell/NA | Can’t Tell/NA | Can’t Tell/NA | Can’t Tell/NA | Can’t Tell/NA | Can’t Tell/NA | Can’t Tell/NA | Can’t Tell/NA | Can’t Tell/NA | Can’t Tell/NA |
| Was the loss to follow-up after baseline 20% or less? Were those lost to follow-up accounted for in the analysis? | Can’t Tell/NA | Can’t Tell/NA | Can’t Tell/NA | Can’t Tell/NA | Can’t Tell/NA | Can’t Tell/NA | Can’t Tell/NA | Can’t Tell/NA | Can’t Tell/NA | Can’t Tell/NA |
| Did the statistical methods examine changes in outcome measures from before to after the intervention? Were statistical tests done that provided p values for the pre-to-post changes? | Yes | Yes | Yes | Yes | Yes | Yes | Yes | Yes | Yes | Yes |
| Were outcome measures of interest taken multiple times before the intervention and multiple times after the intervention? | Yes | Yes | Yes | Yes | Yes | Yes | Yes | Yes | Yes | Yes |
| If the intervention was conducted at a group level did the statistical analysis take into account the use of individual-level data to determine effects at the group level? | Can’t Tell/NA | Can’t Tell/NA | Can’t Tell/NA | Can’t Tell/NA | Yes | Can’t Tell/NA | Can’t Tell/NA | Can’t Tell/NA | Can’t Tell/NA | Can’t Tell/NA |
| **Overall quality rating** | Fair | Poor | Fair | Poor | Good | Poor | Poor | Fair | Good | Fair |

NIH Quality Assessment Tool for the observational cohort and cross-sectional studies

|  | Hoque et al. |
| --- | --- |
| Was the research question or objective in this paper clearly stated? | No |
| Was the study population clearly specified and defined? | Yes |
| Was the participation rate of eligible persons at least 50%? | Can’t Tell/NA |
| Were all the subjects selected or recruited from the same or similar populations (including the same time period)? Were inclusion and exclusion criteria for being in the study prespecified and applied uniformly to all participants? | No |
| Was a sample size justification, power description, or variance and effect estimates provided? | No |
| For the analyses in this paper, were the exposure(s) of interest measured prior to the outcome(s) being measured? | Yes |
| Was the timeframe sufficient so that one could reasonably expect to see an association between exposure and outcome if it existed? | Can’t Tell/NA |
| For exposures that can vary in amount or level, did the study examine different levels of the exposure as related to the outcome (e.g., categories of exposure, or exposure measured as continuous variable)? | No |
| Were the exposure measures (independent variables) clearly defined, valid, reliable, and implemented consistently across all study participants? | Yes |
| Was the exposure(s) assessed more than once over time? | Yes |
| Were the outcome measures (dependent variables) clearly defined, valid, reliable, and implemented consistently across all study participants? | Can’t Tell/NA |
| Were the outcome assessors blinded to the exposure status of participants? | No |
| Was loss to follow-up after baseline 20% or less? | Can’t Tell/NA |
| Were key potential confounding variables measured and adjusted statistically for their impact on the relationship between exposure(s) and outcome(s)? | No |
| **Overall quality rating** | Fair |

Overall quality: Good, Fair, Poor

The questions on the form are designed to help assessors focus on the key concepts for evaluating the internal validity of a study. They are not intended to create a list that assessors simply tally up to arrive at a summary judgment of quality.

Internal validity for cohort studies is the extent to which the results reported in the study can truly be attributed to the exposure being evaluated and not to flaws in the design or conduct of the study–in other words, the ability of the study to draw associative conclusions about the effect of the exposures being studied on outcomes. Any such flaws can increase the risk of bias.

Critical appraisal involves considering the risk of potential for selection bias, information bias, measurement bias, or confounding (the mixture of exposures that one cannot tease out from each other). Examples of confounding include co-interventions, differences at baseline in patient characteristics, and other issues throughout the questions above. High risk of bias translates to a rating of poor quality. Low risk of bias translates to a rating of good quality. (Thus, the greater the risk of bias, the lower the quality rating of the study.)

In addition, the more attention in the study design to issues that can help determine whether there is a causal relationship between the exposure and outcome, the higher quality the study. These include exposures occurring prior to outcomes, evaluation of a dose-response gradient, accuracy of measurement of both exposure and outcome, sufficient timeframe to see an effect, and appropriate control for confounding–all concepts reflected in the tool.

Generally, when assessors evaluate a study, assessors will not see a "fatal aw," but assessors will and some risk of bias. By focusing on the concepts underlying the questions in the quality assessment tool, assessors should ask themselves about the potential for bias in the study they are critically appraising. For any box where assessors check "no" they should ask, "What is the potential risk of bias resulting from this aw in study design or execution?" That is, does this factor cause assessors to doubt the results that are reported in the study or doubt the ability of the study to accurately assess an association between exposure and outcome?

The best approach is to think about the questions in the tool and how each one tells assessors something about the potential for bias in a study. The more assessors familiarize themselves with the key concepts, the more comfortable they will be with critical appraisal. Examples of studies rated good, fair, and poor are useful, but each study must be assessed on its own based on the details that are reported and consideration of the concepts for minimizing bias.
